# Effects of sowing date and nitrogen applications on the energy efficiency of facultative wheat (*Triticum aestivum* L.) in a Pannonian environment

**DOI:** 10.1016/j.heliyon.2024.e37923

**Published:** 2024-09-20

**Authors:** Gerhard Moitzi, Lukas J. Koppensteiner, Agnieszka Klimek-Kopyra, Jaroslav Bernas, Hans-Peter Kaul, Helmut Wagentristl, Pia Euteneuer, Reinhard W. Neugschwandtner

**Affiliations:** a Experimental Farm Groß-Enzersdorf, Department of Crop Sciences, BOKU University, Vienna, 2301 Groß-Enzersdorf, Austria; b Institute of Agronomy, Department of Crop Sciences, BOKU University, Vienna, 3430 Tulln, Austria; c Department of Agroecology and Plant Production, University of Agriculture in Kraków, 31-120 Kraków, Poland; d Department of Agroecosystems, Faculty of Agriculture and Technology, University of South Bohemia, 370 05 České Budějovice, Czech Republic

**Keywords:** Energy efficiency analysis, Facultative wheat, Mineral N fertilization, Sowing date, Pannonian environment

## Abstract

Energy efficiency analysis provides a deeper understanding of non-renewable energy dependent cropping systems. In this study, we examined the crop yield and energy efficiency of facultative rainfed wheat (W_W_ – winter wheat, W_S_ – spring wheat) and mineral nitrogen (N) fertilization (0, 50, 100, 150, and 200 kg N ha^−1^) in two growing seasons 2019/20 and 2020/21 in Central Europe. W_W_ out performed W_S_ significantly overall (2019/20: +30.3 to +47.9 %; 2020/21: +18.9 to +37.3 %) in terms of energy efficiency indicators. The impact of N fertilization on energy efficiency was minimal, largely due to one dose application of mineral N fertilizer. The highest estimated net-energy output (NEO) was observed at 160.2 kg N ha^−1^, which may not sustainable for this pedo-climatic region due to potential N emissions risks. Zero N fertilization showed best performance in terms of energy use efficiency (EUE), energy intensity (EI), and energy productivity (EP). The ERG z-score, which combines NEO and EUE into a single bi-dimensional indicator, indicated an optimal N fertilization level of 72.0 kg N ha^−1^.

## Highlights

• Mineral N fertilization and facultative wheat were evaluated under Pannonian conditions.
• Winter wheat is more energy efficient than spring wheat.
• Mineral N fertilization (0–200 kg N ha^−1^) has a low effect on energy efficiency.
• Optimization of N fertilization was estimated with 72 kg N ha^−1^.
• Crop yield determines mainly the energy efficiency indicators.

## 1 Introduction

Agriculture plays a central role in the European Green Deal, with various policies and strategies converging to promote sustainable agriculture and food systems. The European Green Deal, which proposes to achieve a climate-neutral Europe by 2050, strongly focusing on the transition to a more sustainable agriculture and food system to contribute to climate change mitigation and biodiversity protection [1]. The European Commission wants to reduce emissions by 2025 b y 50–55 % compared to the 1990's and the Farm to Fork strategy aims to ensure that agriculture, fisheries, aquaculture and food chain contributes significantly to this target [1]. It proposes to achieve a minimum of 25 % organic farming in Europe, to reduce the use of pesticides by 50 % and the use of fertilizers by 20 %, all in less than a decade (by 2030).

Energy is either used directly as fuel or electricity to operate machinery and equipment or indirectly as fertilizers, pesticides, seeds and farm machinery [2]. As arable farming has become increasingly mechanized, it requires significant energy inputs at particular stages of the production cycle to achieve optimum yields. The efficient use of N fertilizers will become a key driver for sustainable agricultural systems in the future [3] and increases the energy efficiency in plant production [4,5]. N losses from the soil-plant system lower the nitrogen use efficiency and are not sustainable for economic and ecological reasons.

The optimal management of cropping systems (e.g. by fertilization and tillage) is a key for high energy efficiency [4,6]. Conventional high-input cropping systems maximize crop yield and energy output, whereas high energy use efficiency is found in organic crop management [7]. According to a case study in northeast Italy, the mean crop yield in organic farming was 31 % (range over the years from 10 % to 55 %) lower than in conventional farms. But the energy use efficiency was higher in organic farming due to the reduced energy input, mainly fertilization [8].

Wheat (*Triticum aestivum* L.) was grown in Austria in 2021 on 279 860 ha with a mean yield of 5530 kg ha^−1^. In Europe, about 62.8 million ha wheat was grown in 2021 with a mean yield of 4285 kg ha^−1^ [9]. Production of wheat is affected by changing weather conditions, which are difficult to predict, especially under climate change. Therefore, facultative crops can be beneficial, because of their high adaptability in sowing time [10]. Shifting the sowing date, e.g. from spring to autumn, can reduce heat and drought stress in summer. Under Pannonian climate conditions, autumn-sowing has, compared to spring-sowing, resulted in higher yields of faba bean, pea, wheat, and triticale [[10], [11], [12]]. Also, the intensity of nutrient input (N mainly) and intensity of all the inputs during the vegetation period play a role in the overall environmental impact [13]. From the point of sowing date and N fertilization of facultative wheat in Pannonian condition a comprehensive energy analysis is a research gap. Consequently, we evaluated in this study the energy efficiency of facultative wheat having winter wheat and spring wheat in different mineral N fertilization levels (0, 50, 150, 200 kg N ha^−1^) in two growing seasons in the pedo-climatic region of Pannonia. The main objectives were (i) to identify the effect of facultative wheat and mineral N fertilization on energy efficiency and (ii) to estimate the optimal mineral N fertilization for the best energy efficiency performance.

## 2 Materials and methods

The management data (tillage, plant protection, fertilization) and the crop yield data of facultative wheat were collected from a two-factorial field trial for two growing seasons and transformed to a 1 ha base unit.

### 2.1 Experimental site and climatic data

The experiment was carried out in the growing seasons 2019/20 and 2020/21 at the Experimental Station of the BOKU University, Vienna, in Raasdorf (48°14′N, 16°33′E; 153 m a.s.l.). The field is located east of Vienna, Austria, on the edge of the Marchfeld Plain, which is an important crop production region in the north-western part of the Pannonian Basin. The silty loam soil (pH_CaCl2_: 7.6, soil organic carbon: 2.3 %) is classified as a Calcaric Chernozem of alluvial origin [14]. The mean, maximum and minimum values in the wheat growing season (October–July) for temperature were: 9.3 °C, 11.9 °C, 7.4 °C and for the precipitation: 425.2 mm, 606.0 mm, 320.5 mm (weather station Groβ-Enzersdorf; observation period: 1980–2018). The temperature in February and March was higher in 2019/20, whereas it was lower in March as well as May in 2020/21 compared to the long-term mean (Table 1). In both seasons, the amount of precipitation was lower in March and April and higher in May compared to the long-term data in both seasons. In June, rainfall was higher in 2019/20 and lower in 2020/21 [15].

**Table 1:** Mean precipitation and mean temperature during growing seasons and long-term values.

	Precipitation (mm)			Temperature (°C)		
	1980–2018	2019/20	2020/21	1980–2018	2019/20	2020/21
October	37.0	15.9	90.5	10.9	12.6	11.7
November	36.7	56.2	16.2	5.5	8.2	6.3
December	31.9	31.7	27.3	1.4	3.5	3.5
January	26.1	18.5	23.7	0.3	1.0	1.7
February	25.2	23.1	13.1	1.8	7.0	2.5
March	34.8	25.2	7.3	6.1	7.3	5.8
April	37.3	8.8	14.5	11.0	11.8	9.2
May	60.0	81.3	82.2	15.7	14.5	14.0
June	70.4	118.6	22.4	18.9	19.3	21.6
July	65.8	54.2	112.9	21.2	21.4	22.9
**October - July**	**425.2**	**433.5**	**410.1**	**9.3**	**10.7**	**9.9**

### 2.2 Experimental design and management

The experiment was conducted in a split-plot design with the two factors sowing time (W_W_ – winter wheat, W_S_ – spring wheat) and N fertilization (50, 100, 150 and 200 kg N ha^−1^, supplemented by a zero N (0 kg N ha^−1^) as control). The main-plot factor sowing time was established in complete blocks, and the sub-plot factor N fertilization was completely randomized among subplots within the main plots. Three replications were performed. The pre-crop was in both years winter barley. A reduced tillage system with cultivator was applied: Primary tillage was carried out with a wing sweep cultivator to a depth of 20 cm. The wing sweep cultivator had seven tines on 2 bars with a tine distance of 84 cm and a line distance of 42 cm. Three rotary hoes for crumbling and a wedge ring roller for crumbling and depth adjustment were mounted behind the tine bars. After secondary tillage with a power harrow, seeding of facultative wheat (cv. Lennox) with 300 germinable seeds m^−2^ was done of W_W_ on October 17, 2019 and October 27, 2020 and of S_W_ on March 10, 2020 and March 8, 2021 with an Oyjard plot drill seeder at a depth of 4 cm. This resulted in a different thousand kernel weight in a sowing rate of 106 kg ha^−1^ in 2019/20 and 131 kg ha^−1^ in 2020/21. The plot size was 36 m^2^ (12 × 3 m) with a row spacing of 12.5 cm. The soil mineral nitrate (NO_3_-N) contents in 0–0.9 m depth at sowing were 34.0 kg ha^−1^ (autumn 2019), 58.7 kg ha^−1^ (spring 2020), 60.4 kg ha^−1^ (autumn 2020) and 72.1 kg ha^−1^ (spring 2021).

The stabilized urea fertilizer ALZON 46® (46 % N) was applied manually with five rates (0, 50, 100, 150 and 200 kg ha^−1^) on March 10, 2020 and on March 10, 2021 to the soil surface without incorporation into the soil. Plants were sprayed against weeds (5 g a.i. ha^−1^ of florasulam and 45 g a.i. ha^−1^ of pinoxaden (Axial komplett®) in April 2020; 20 g a.i. ha^−1^ of carfentrazone-ethyl, 700 g a.i. ha^−1^ of mecoprop-P and 320 g a.i. ha^−1^ of 2,4-D (Aurora 40 WG® and Duplosan KV neu®) in May 2021), fungal diseases (125 g a.i. ha^−1^ of prothioconazol and 125 g a.i. ha^−1^ of tebuconazol (Prosaro®) in May 2020 and 2021) and pests (7.5 g a.i. ha^−1^ of deltamethrin (Decis Forte®) in May 2020 and 2021).

Harvest was conducted in 2020 on 6 July (W_W_) and 20 July (W_S_) and in 2021 on 13 July (W_W_) and 26 July (W_S_). Samples were cut manually at the soil surface, divided into grains and straw and dried at 105 °C for 24 h. For comparison to other studies and results in practical farming, the yield was recalculated to a moisture content of 14 %.

### 2.3 Fuel consumption

Fuel consumption for primary tillage, seedbed preparation and seeding were recorded on a neighbouring field. A high-performance flow-meter (PLU 116H; AVL®, Graz, Austria) with a proportional integral (PI) controller was integrated into the fuel system of a 92 kW and a 59 kW four-wheel-drive tractor (Steyr 9125 and Steyr 8090, St. Valentin, Austria). Fuel consumption for stubble cultivation was recorded by the tractor terminal (Datatronic 5) of the 200 kW four-wheel-drive tractor (Massey Ferguson 8S.265, AGCO Corporation, Beauvais Cedex, France). The soil was dry during tillage with a mean gravimetric soil water content of 15 % in a 0–20 cm layer. Data for fuel consumption of the sprayer (2 L ha^−1^ for one process), the fertilizer spreader (1.5 L ha^−1^ for one process) and the combine harvester (25 L ha^−1^ for one process) were obtained from the Austrian Association for Agricultural Engineering and Landscape Development [16]. Pesticide applications were conducted with two passes in the year 2020 and one pass in the year 2021. For transportation of the harvested grain, fuel consumption was calculated with a specific fuel consumption coefficient of 0.09 L (L) fuel per ton of wheat and kilometre [16]. A distance of 5 km for the transportation of grain with a tractor and a trailer was assumed. Two percent of the fuel consumption was assumed for the lubrication oil of motors [17].

### 2.4 Energy analysis

We adopted a process analysis methodology in which support energy (energy input for crop production) and outputs were traced following physical material flows [18,19]. The material flows were transformed into energy flows by multiplying material flows with specific energy equivalents (Table 3). Only direct and indirect consumption of non-renewable energy resources, which are modified by agricultural management, were considered [19].

Direct energy refers to the energy content of fuel and lubricants, whereas indirect energy counts for the production of seed, fertilizer, pesticides and farm machinery [[18], [19], [20], [21]]. The environmental inputs (e.g. solar radiation, precipitation, water, wind) and labour inputs were not directly considered. Solar radiation as a high input is considered indirectly in the photosynthetic above-biomass production. Labour energy can be accounted for in the energy analysis as input [22], but we did not consider it because it is usually insignificant compared to other inputs [19,23]. We used different indicators to analyze the energy efficiency of wheat production (Table 2) [18,[24], [25], [26]]. Energy input and energy output were combined to calculate net-energy output, energy intensity, energy productivity and energy use efficiency. These parameters were mainly determined by the higher energy output in comparison to the lower energy input [25,27]. The boundary of the energy analysis is the field gate with a 5 km road distance from the field to the farm. That means the fuel energy input for the on-road drives of the tractor and combine harvester was considered in the energy analysis. The energy consumption, which is not related to crop management techniques, e.g. energy in farm buildings, grain drying and conservation and the rest of the food chains, was not considered [19].

**Table 2:** Definition of energy efficiency indicators.

Parameter	Definition	Unit
**Energy input (E)**		
Direct energy input (E_d_)	E_d_ = diesel fuel and lubricant oil	MJ ha^−1^
Indirect energy input (E_i_)	E_i_ = seed + fertilizer + pesticide + machines	MJ ha^−1^
Total energy input (E)	E = E_d_ + E_i_	MJ ha^−1^
**Energy output (EO)**		
EO_GRAIN_	EO_GRAIN_ = grain yield × gross energy in grain	GJ ha^−1^
EO_STRAW_	EO_STRAW_ = straw yield × gross energy in straw	GJ ha^−1^
EO_AGB_^aa^	EO_AGB_ = EO_GRAIN_ + EO_STRAW_	GJ ha^−1^
**Net-energy output (NEO)**		
NEO	NEO = EO_AGB_ – E	GJ ha^−1^
**Energy use efficiency (EUE)**		
EUE_GRAIN_	EUE_GRAIN_ = EO_GRAIN_/E × 1000	GJ GJ^−1^
EUE_AGB_	EUE = EO_AGB_/E × 1000	GJ GJ^−1^
**Energy productivity (EP)**		
EP_GRAIN_	EP_GRAIN_ = grain yield^B^/E	kg MJ^−1^
EP_STRAW_	EP_STRAW_ = straw yield^B^/E	kg MJ^−1^
EP_AGB_	EP_AGB_ = AGB yield^B^/E	kg MJ^−1^
**Energy intensity (EI)**		
EI_GRAIN_	EI_GRAIN_ = E/grain yield^B^	MJ kg^−1^
EI_AGB_	EI_AGB_ = E/AGB yield^B^	MJ kg^−1^
Grain allocated EI (EI_GRAIN_)	allocated EI_GRAIN_ = E/AGB yield^B^ × HI^C^/100	MJ kg^−1^
Straw allocated EI (EI_STRAW_)	allocated EI_STRAW_ = E/AGB yield^b^ × (1 – HI)/100	MJ kg^−1^
**Fuel intensity (FI)**		
FI	FI = diesel fuel/grain yield^B^ × 100	L 100 kg^−1^

a Above-ground biomass.

b 14 % moisture content,^c^ Harvest index.

**Table 3:** Direct and indirect energy equivalents used in this study.

	Unit	Energy equivalent	References
**Direct energy use**			
Diesel fuel	MJ L^−1^	39.6	[18,30]
Lubricant oil	MJ L^−1^	39.0	[31]
**Indirect energy use**			
**Mineral fertilizer**			
Stabilized Urea (46 % N)	MJ N kg^−1^	41.7	[32]
**Pesticide**			
Herbicide for broadleaf weed control	MJ kg^−1^	259	[33]
Insecticide (Deltamethrin - Decis)	MJ kg^−1^	217	[34]
Fungicide	MJ kg^−1^	180	[33]
**Seed**			
Winter wheat	MJ kg^−1^	5.5	[18]
**Machinery**^**1)**^			
Conservation tillage	MJ ha^−1^	1810	[29]

Energy in wheat grain was set to 18.6 MJ kg^−1^ dry matter and energy in wheat straw was set to 18.2 MJ kg^−1^ dry matter [18], which is similar to the gross energy content in feed wheat [28]. The area-specific embedded energy in farm machinery was calculated for the winter wheat production in a conservation tillage system with a cultivator based on a 100 ha arable area under Austrian conditions [29]. The estimated technical and economic life was set to 10000 h for the tractor, 3000 h for the combine harvester, 3000 ha for the short disc harrow, cultivator and power harrow, 2100 ha for the seed drill, 2000 t for the two-disc spreader, 6000 ha for the sprayer and 63000 t for the trailer.

The EI was calculated by the amounts of the used input facilities (fuel, fertilizer, pesticides, seeds) multiplied by the energy equivalents (Table 3).

The optimum rate of N fertilization is different for NEO and EUE [18,35]. Optimizing of mineral fertilizer rates have mainly focused on maximizing crop yield (corresponding with NEO) or economic profitability [35]. Optimization, according to EUE implies that more land will be needed to compensate for the yield reduction caused by the relatively low input of fertilizer [4,35]. Due to incompatible targets of NEO and EUE, Rossner et al. [35] recommended a bi-dimensional optimization approach by consideration of both NEO and EUE. In our study, we used this z-score approach by combining NEO with EUE: NEO and EUE were standardised as (x – mean)/SD. The combined index, referred to ERG z-index is calculated as the sum of the two standardised measures for NEO and EUE, which is used afterwards for the calculation of the optimum N fertilization level (N_OPT_) [35]. Mean, and SD (standard deviation) were calculated for each year × sowing time combination.

### 2.5 Statistical analysis

All statistical analyses were conducted using IBM® SPSS® Statistics 21. The requirements for analysis of variance (ANOVA) were tested with the Levene test for homogeneity of variances and the Shapiro-Wilk test for normal distribution of residuals. Two-factorial ANOVA tests were carried out for the dependent variables (crop yields, harvest index and energy efficiency indicators) to detect sowing time (S) and N fertilization (N) effects. Multiple comparisons to separate means were carried out with the Student-Newman-Keuls procedure (p < 0.05).

The significant differences among coefficients of second order polynomials, which include intercepts, linear coefficients and quadratic coefficients were tested with the Student-Newman-Keuls procedure (p < 0.05). The dependent variables were regressed on the rate of mineral N using a quadratic function (y = a + b_1_x + b_2_x^2^). The rates of mineral N (N_OPT_) for achieving maximum crop yields, EO, NEO and ERG z-index were estimated by putting the first derivative of the quadratic function equal to 0 and then solving the equation [35]. The goodness of fit of the equation was indicated by the coefficient of determination (R^2^).

## 3 Results

### 3.1 Fuel consumption and energy input

The area-based fuel consumption in the growing season 2019/20 in the N fertilized treatments was 2.6 % higher than in the control (Table 4). As the pesticide application was done in 2019/20 twice and in 2020/21 just one time, the area-based fuel consumption was in the control by 3.6 % higher and in the N fertilized treatments by 3.5 % higher in 2019/20 than in 2020/21.

**Table 4:** Diesel fuel consumption (L ha^−1^) for facultative wheat production in the season 2019/20 and 2020/21.

Operation	N fertilization (kg N ha^−1^)	
	0	50, 100, 150, 200
Stubble cultivation^a^ Tractor (200 kW) pulled short disc harrow (6 m)	5.7	5.7
Primary tillage^b^ Wing sweep cultivator (3 m), 92 kW	9.4	9.4
Seedbed preparation^c^ Power harrow (3 m), 75 kW	8.6	8.6
Seeding^c^ Universal seed drill (3 m), 75 kW	6.3	6.3
Spreading fertilizer^d^ Two-disc spreader (12–45 m), 57 kW	–	1.5
Spraying herbicide^d^, Pulled sprayer (21 m), 57 kW, two passes in 2019/20 and one pass 2020/21	4.0/2.0	4.0/2.0
Harvesting^d^ Combine harvester (5 m), 200 kW	22.0	22.0
Transport (5 km)^d^ Two-axle trailer (19 m^3^), 92 kW	1.8	1.8
**Total**	**57.8/55.8**	**59.3/57.3**

a measured in July 2022, unpublished.

b [36].

c [37].

d [16].

The calculated total energy input ranged between 4998 MJ ha^−1^ and 13394 MJ ha^−1^ in 2019/20 and between 5136 MJ ha^−1^ and 13532 MJ ha^−1^ in 2020/21 (Table 5). The ratios of direct energy to indirect energy (in %:%) were for the N fertilization treatments and over both years: 46:54 for 0 kg N ha^−1^, 33:67 for 50 kg N ha^−1^, 26:74 for 100 kg N ha^−1^, 21:79 for 150 kg N ha^−1^ and 18:82 for 200 kg N ha^−1^. The share of fertilizer energy on the total energy input was at 29 % (50 kg N ha^−1^), 45 % (100 kg N ha^−1^), 55 % (150 kg N ha^−1^) and 62 % (200 kg N ha^−1^). The energy input that was allocated to machinery contributed between 36 % (0 kg N ha^−1^) and 14 % (200 kg N ha^−1^) to the total energy input. Between 5 % (200 kg N ha^−1^) and 13 % (0 kg N ha^−1^) of the total energy input was allocated to seeds. The lowest energy input share had the herbicides with about 2 % (200 kg N ha^−1^) to 5 % (0 kg N ha^−1^).

**Table 5:** Mean direct and indirect energy input (MJ ha^−1^) for facultative wheat production in different mineral N fertilization.

	N fertilization (kg N ha^−1^)				
	0	50	100	150	200
**Direct energy**					
Fuel, lubricant	2339	2395	2395	2395	2395
**Indirect energy**					
Seed^a^ (2019/20/2020/21)	583/721	583/721	583/721	583/721	583/721
Fertilizer	0	2085	4170	6255	8340
Herbicide	266	266	266	266	266
Machinery	1810	1810	1810	1810	1810
**Total**^A^ (2019/20/2020/21)	4998/5136	7139/7277	9224/9362	11309/11447	13394/13532

a Seed amount differed between years. This also affected the total energy input in the two years. All other parameters were set fixed for both years.

### 3.2 Crop yield and energy output

The mean crop yields, harvest indices and energy output parameters (overall sowing times and N fertilization treatments) were in 2019/20 or 2020/21 for grain yield: 5199 or 4715 kg ha^−1^, straw yield: 4912 or 6909 kg ha^−1^, harvest index: 51.3 or 40.5 %, EO_GRAIN_: 84.8 or 76.9 GJ ha^−1^, EO_STRAW_: 78.4 or 110.3 GJ ha^−1^, EO_AGB_: 163.2 or 187.2 GJ ha^−1^ (Table 6). Consequently, mean values were in 2019/20 higher or lower than in 2020/21 for grain yield: +10.3 %, straw yield: 28.9 %, harvest index: +26.7 %, EO_GRAIN_: +10.3 %, EO_STRAW_: 28.9 % and EO_AGB_: +12.8 %. Values for main factors and their factor levels are shown in Table 6.

**Table 6:** Crop yield, harvest index and energy output (EO), affected by sowing time and N fertilization in 2019/20 and 2020/21.

	Grain yield^a^ (kg ha^−1^)		Straw yield^a^ (kg ha^−1^)		Harvest index (%)		EO_GRAIN_ (GJ ha^−1^)		EO_STRAW_ (GJ ha^−1^)		EO_AGB_ (GJ ha^−1^)	
	2019/20	2020/21	2019/20	2020/21	2019/20	2020/21	2019/20	2020/21	2019/20	2020/21	2019/20	2020/21
Sowing time (T)												
W_W_	6196^b,^^b^	5455^b^	5760^b^	7676^b^	51.7	41.6	101.1^b^	89.0^b^	92.0^b^	122.6^b^	193.0^b^	211.6^b^
W_S_	4202^a^	3974^a^	4064^a^	6142^a^	50.9	39.5	68.6^a^	64.8^a^	64.9^a^	98.1^a^	133.4^a^	162.9^a^
N fertilization (N)												
0	4068^a^	3942	3920^a^	4982^a^	51.0	44.3	66.4^a^	64.3	62.6^a^	79.5^a^	129.0^a^	143.9^a^
50	5009^b^	4687	4791^b^	6934^b^	51.3	39.9	81.7^b^	76.5	76.5^b^	110.7^b^	158.2^b^	187.2^b^
100	5439^b^	4912	5085^b^	7453^b^	51.6	39.4	88.7^b^	80.1	81.2^b^	119.0^b^	170.0^b^	199.1^b^
150	5790^b^	5034	5373^b^	7575^b^	51.7	39.8	94.5^b^	82.1	85.8^b^	120.9^b^	180.3^b^	203.1^b^
200	5689^b^	4999	5390^b^	7601^b^	51.1	39.2	92.82^b^	81.6	86.0^b^	121.4^b^	178.9^b^	202.9^b^
ANOVA												
T	∗∗∗	∗∗∗	∗∗∗	∗∗∗	ns	ns	∗∗∗	∗∗∗	∗∗∗	∗∗∗	∗∗∗	∗∗∗
N	∗∗∗		∗∗	∗∗∗	ns		∗∗∗		∗∗	∗∗∗	∗∗∗	∗∗∗
N × T	∗	ns	ns	∗	ns	ns	∗	ns	ns	∗	ns	ns

a 14 % moisture content.

b Statistically significant differences within factors T and N are shown with small letters. Significance level: ns = not significant, p < 0.05 (∗), p < 0.01 (∗∗), p < 0.001 (∗∗∗).

The mean grain yield and mean EO_GRAIN_ (overall N fertilization treatments) were higher in W_W_ than in W_S_ (by a mean of +47.5 % in 2019/20 and + 37.3 in 2020/21). The mean straw yield and EO_STRAW_ were higher in W_W_ than in W_S_ (by a mean of +41.7 % in 2019/20 and + 25.0 % in 2020/21). The mean EO_AGB_ (overall N fertilization treatments) was in W_W_ by 44.7 % in 2019/20 and by 29.9 % in 2020/21, higher than in W_S_. N fertilization increased the straw yield and EO_STRAW_ in 2019/20 and the EO_AGB_ in both years, but no statistical differences were observed for these parameters between the N fertilization doses (50, 100, 150, 200 kg N ha^−1^).

The harvest index was not affected by sowing time and N fertilization but tended to be higher in the control than in fertilized treatments in 2020/21 (p = 0.052).

The detected significant sowing time × N fertilization interaction in 2019/20 for the grain yield and EO_GRAIN_ and in 2020/21 for the straw yield and EO_STRAW_ show an increase with higher N rates, which levelled off at a lower N rate for W_S_ compared to W_W._

### 3.3 Energy efficiency indicator

The mean values of energy indicators for net-energy output, energy use efficiency and energy productivity (overall sowing times and N fertilization treatments) were in 2019/20 or 2020/21 for NEO: 152.2 or 177.7 GJ ha^−1^, EUE_GRAIN_: 10.2 or 8.7 GJ GJ^−1^, EUE_AGB_: 19.7 or 21.0 GJ GJ^−1^), EP_GRAIN_: 0.63 or 0.53 kg MJ^−1^, EP_STRAW_: 0.59 or 0.77 kg MJ^−1^, EP_AGB_: 1.22 or 1.30 kg MJ^−1^. Consequently, mean values were in 2019/20 higher or lower than in 2020/21 for NEO: 15.2 %, EUE_GRAIN_: +14.7 %, EUE_AGB_: 6.6 %, EP_GRAIN_: +15.9 %, EP_STRAW_: 30.5 % and EP_AGB_: 6.6 %. Values for the main factors and their factor levels are shown in Table 7. No statistically significant interactions between sowing dates and N fertilization were observed.

**Table 7:** Net-energy output (NEO), energy use efficiency (EUE), energy productivity (EP) affected by sowing time and N fertilization in 2019/20 and 2020/21.

	NEO (GJ ha^−1^)		EUE_GRAIN_ (GJ GJ^−1^)		EUE_AGB_ (GJ GJ^−1^)		EP_GRAIN_ (kg MJ^−1^)		EP_STRAW_ (kg MJ^−1^)		EP_AGB_ (kg MJ^−1^)	
	2019/20	2020/21	2019/20	2020/21	2019/20	2020/21	2019/20	2020/21	2019/20	2020/21	2019/20	2020/21
Sowing time (T)												
W_W_	184.0^b,1^	202.0^b^	12.0^b^	9.9^b^	23.0^b^	23.4^b^	0.73^b^	0.61^b^	0.69^b^	0.84^b^	1.43^b^	1.45^b^
W_S_	124.4^a^	153.3^a^	8.4^a^	7.5^a^	16.3^a^	18.6^a^	0.52^a^	0.46^a^	0.50^a^	0.69^a^	1.01^a^	1.15^a^
N fertilization (N)												
0	124.2^a^	138.5^a^	13.9^e^	12.0^d^	26.9^e^	26.9^c^	0.85^e^	0.74^d^	0.82^d^	0.93^c^	1.67^e^	1.67^c^
50	151.3^b^	179.7^b^	11.8^d^	10.2^c^	22.8^d^	25.0^c^	0.72^d^	0.63^c^	0.69^c^	0.93^c^	1.41^d^	1.55^c^
100	160.9^b^	189.6^b^	9.8^c^	8.4^b^	18.8^c^	20.8^b^	0.60^c^	0.51^b^	0.56^b^	0.78^b^	1.17^c^	1.29^b^
150	169.1^b^	191.4^b^	8.5^b^	7.0^ab^	16.2^b^	17.4^a^	0.52^b^	0.43^ab^	0.48^ab^	0.65^a^	1.01^b^	1.08^a^
200	165.7^b^	189.2^b^	7.0^a^	5.9^a^	13.6^a^	14.8^a^	0.43^a^	0.36^a^	0.41^a^	0.55^a^	0.84^a^	0.92^a^
ANOVA												
T	∗∗∗	∗∗∗	∗∗∗	∗∗∗	∗∗∗	∗∗∗	∗∗∗	∗∗∗	∗∗∗	∗∗∗	∗∗∗	∗∗∗
N	∗∗	∗∗∗	∗∗∗	∗∗∗	∗∗∗	∗∗∗	∗∗∗	∗∗∗	∗∗∗	∗∗∗	∗∗∗	∗∗∗
N × T	ns	ns	ns	ns	ns	ns	ns	ns	ns	ns	ns	ns

^1)^ Significant differences within factors T and N are shown with small letters. Significance level: ns = not significant, p < 0.05 (∗), p < 0.01 (∗∗), p < 0.001 (∗∗∗).

W_W_ performed significantly better than W_S_. The energy efficiency parameters were in 2019/20 or 2020/21 higher for NEO by 47.9 % or 31.8 %, EUE_GRAIN_ by 42.9 % or 32.0 %, EUE_AGB_ by 41.1 % or 25.8 %, EP_GRAIN_ by 40.4 % or 32.6 %, EP_STRAW_ by 38.0 % or 21.7 % and EP_AGB_ by 41.6 % or 26.1 %.

The lowest NEO values but generally the highest EUE and EP values were found in the control. N fertilization significantly affected energy efficiency indicators, except for EUE_AGB_, EP_STRAW_ and EP_AGB_ in 2020/21, and no significant differences occurred between 0 and 50 kg N ha^−1^. The NEO was in both years higher with all fertilization doses compared to the control with no differences between N doses. Ranking among fertilization treatments (in kg N ha^−1^) was in 2019/20 as follows for EUE_GRAIN_, EUE_AGB_, EP_GRAIN_ and EP_AGB_: 0 > 50 > 100 > 150 > 200 and for EP_STRAW_: 0 > 50 > 100 ≥ 150 ≥ 200. The ranking was in 2020/21 as follows for EUE_GRAIN_ and EP_GRAIN_: 0 > 50 > 100 ≥ 150 ≥ 200 and for EUE_AGB_, EP_RES_ and EP_AGB_: 0 and 50 > 100 > 150 and 200.

The mean values of intensity indicators for energy and fuel (overall sowing times and N fertilization treatments) were in 2019/20 or 2020/21 for EI_GRAIN_: 1.78 or 2.08 MJ kg^−1^, EI_AGB_: 0.91 or 0.83 MJ kg^−1^, allocated EI_GRAIN_: 0.47 or 0.33 MJ kg^−1^, allocated EI_STRAW_: 0.44 or 0.50 MJ kg^−1^, FI: 1.20 or 1.31 L 100 kg^−1^. Consequently, mean values were in 2019/20 higher or lower than in 2020/21 for EI_GRAIN_: 16.9 %, EI_AGB_: +8.8 %, allocated EI_GRAIN_: 29.8 %, allocated EI_STRAW_: 13.6 % and FI: 9.2 %. Values for the main factors and their factor levels are shown in Table 8.

**Table 8:** Energy intensity (EI) and fuel intensity (FI) affected by sowing time and N fertilization in seasons 2019/20 and 2020/21.

	EI_GRAIN_ (MJ kg^−1^)		EI_AGB_ (MJ kg^−1^)		Allocated EI_GRAIN_ (MJ kg^−1^)		Allocated EI_STRAW_ (MJ kg^−1^)		FI (L 100 kg^−1^)	
	2019/20	2020/21	2019/20	2020/21	2019/20	2020/21	2019/20	2020/21	2019/20	2020/21
Sowing time (T)										
W_W_	1.43^a,^^a^	1.76^a^	0.74^a^	0.72^a^	0.38^a^	0.30^a^	0.36^a^	0.42^a^	0.99^a^	1.12^a^
W_S_	2.13^b^	2.41^b^	1.08^b^	0.94^b^	0.55^b^	0.37^b^	0.53^b^	0.57^b^	1.42^b^	1.50^b^
N fertilization (N)										
0	1.19^a^	1.37^a^	0.61^a^	0.61^a^	0.31^a^	0.27^a^	0.30^a^	0.34^a^	1.44^b^	1.48
50	1.44^b^	1.67^ab^	0.74^b^	0.66^a^	0.38^ab^	0.26^a^	0.36^b^	0.40^a^	1.23^a^	1.32
100	1.72^c^	2.06^bc^	0.88^c^	0.80^b^	0.45^b^	0.31^b^	0.43^c^	0.49^b^	1.13^a^	1.28
150	2.04^d^	2.39^c^	1.05^d^	0.95^c^	0.54^c^	0.38^c^	0.51^d^	0.57^d^	1.09^b^	1.22
200	2.49^e^	2.94^d^	1.26^e^	1.13^d^	0.64^d^	0.44^d^	0.62^e^	0.69^d^	1.12^b^	1.27
ANOVA										
T	∗∗∗	∗∗∗	∗∗∗	∗∗∗	∗∗∗	∗∗∗	∗∗∗	∗∗∗	∗∗∗	∗∗∗
N	∗∗∗	∗∗∗	∗∗∗	∗∗∗	∗∗∗	∗∗∗	∗∗∗	∗∗∗	∗∗∗	ns
N × T	∗∗	ns	∗	∗	ns	∗	∗∗	ns	ns	ns

a Significant differences within factors T and N are shown with small letters. Significance level: ns = not significant, p < 0.05 (∗), p < 0.01 (∗∗), p < 0.001 (∗∗∗).

W_W_ showed significantly lower intensity indicators than W_S_: EI_GRAIN_ in 2019/20 or 2020/21: 32.9 % or −27.0 %, EI_AGB_: 31.5 % or −23.4 %, allocated EI_GRAIN_: 30.9 % or −18.9 %, allocated EI_STRAW_: 32.1 % or −26.3 %, FI: 30.3 % or −25.3 %.

The lowest energy intensity and highest fuel intensity were found in the zero N fertilization. The energy intensity increased significantly with increasing N fertilization, whereas the fuel intensity showed a lower response to N fertilization (Table 8).

For the significant sowing time × N fertilization interactions (Table 8), steady increases with higher N rates for both sowing times were observed, but the absolute increases were substantially larger with W_S_.

### 3.4 Curve estimation for crop yield and energy output

The coefficients for the quadratic equations with R^2^ and N_OPT_ are shown in Table 9. The overall estimated dependent variables for the control were (over both years) for grain yield: 4035 kg ha^−1^, straw yield: 4545 kg ha^−1^, harvest index: 47.4 %, EO_GRAIN_: 65.8 GJ ha^−1^, EO_STRAW_: 72.5 GJ ha^−1^, EO_AGB_: 138.4 GJ ha^−1^.

**Table 9:** Coefficient R^2^, estimated optimum N fertilization level (N_OPT_) for maximum crop yield, harvest index and EO with the coefficient of the second order polynomial for N fertilization.

	R^2^	N_OPT_	Intercept	Linear	Quadratic
Grain yield (kg ha^−1^) and EO_GRAIN_ (GJ ha^−1^)			Grain yield/EO_GRAIN_	Grain yield/EO_GRAIN_	Grain yield/EO_GRAIN_
W_W_-2019/20	0.749∗∗∗	180.1	4505.760/73.515	29.082^b^/0.475^b^	−0.081211/-0.001325
W_S_-2019/20	0.473 (p = 0.021)∗	144.6	3665.408/59.804	11.728^ab^/0.191^ab^	−0.042397/-0.000692
W_W_-2020/21	0.435 (p = 0.032)∗	168.9	4172.156/68.072	27.200^b^/0.444^b^	−0.095796/-0.001563
W_S_-2020/21	0.097	28.5	3797.336/61.957	1.657^a^/0.027^a^	0.000757/0.000012
Mean		130.5 (164.5)^a^			
Straw yield (kg ha^−1^) and EO_STRAW_ (GJ ha^−1^)			Straw yield/EO_STRAW_	Straw yield/EO_STRAW_	Straw yield/EO_STRAW_
W_W_-2019/20	0.601∗∗	178.7	4478.574^ab^/71.500^ab^	23.197^a^/0.370^a^	−0.069223^ab^/-0.001105
W_S_-2019/20	0.379 (p = 0.057)	211.2	3446.924^a^/55.030^a^	10.481^a^/0.167^a^	−0.028754^b^/-0.000459
W_W_-2020/21	0.859∗∗∗	167.0	5280.891^b^/84.309^b^	45.138^b^/0.721^b^	−0.141225^a^/-0.002255
W_S_-2020/21	0.592 (p = 0.005)∗	135.9	4971.716^b^/79.373^b^	26.934^a^/0.430^a^	−0.101532^ab^/-0.001621
Mean		173.2 (160.5)^a^			
Harvest index (%)		173.2	Harvest index	Harvest index	Harvest index
W_W_-2019/20	0.443∗	159.6	50.298^b^	0.021^b^	−0.000043^a^
W_S_-2019/20	0.224	70.4	51.534^b^	0.004^b^	−0.000067^a^
W_W_-2020/21	0.242	28.1	43.955^a^	−0.031^b^	0.000053^b^
W_S_-2020/21	0.385	123.8	43.696^a^	−0.108^a^	0.000441^b^
Mean		95.5			
EO_AGB_ (GJ ha^−1^)			EO_AGB_	EO_AGB_	EO_AGB_
W_W_-2019/20	0.692∗∗∗	174.6	145.015	0.845^ab^	−0.002430
W_S_-2019/20	0.425∗	167.6	114.834	0.359^a^	−0.001151
W_W_-2020/21	0.737∗∗∗	164.9	152.381	1.164^b^	−0.002255
W_S_-2020/21	0.513 (p = 0.013)∗	203.7	141.330	0.457^a^	−0.001609
Mean		177.7			

a The mean values just for significant interactions are in brackets. Significance level for R^2^: p < 0.05 (∗), p < 0.01 (∗∗), p < 0.001 (∗∗∗).

The estimated curves for the overall dataset showed for grain yield (Fig. s1) and EO_GRAIN_ (not shown) as well as for straw yield (Fig. s2) and EO_STRAW_ (not shown) and EO_AGB_ (Fig. s3) a degressive increase to an estimated N_OPT_ (Table 9). N_OPT_ for grain yield (and EO_GRAIN_), straw yield (and EO_STRAW_) and EO_AGB_ were estimated at 164.4 kg N ha^−1^, 160.5 kg and 177.7 kg N ha^−1^, respectively (Table 9).

### 3.5 Curve estimation for energy efficiency indicators

For the energy efficiency indicators NEO, FI and ERG z-index a point of inflection was identified within the range of 0 and 200 kg N ha^−1^ (Fig. s4, s14, s15), whereas the other energy indicators revealed no point of inflection (Figure s5 – s11). Consequently, an overall optimum N fertilization level (N_OPT_) could be found for NEO with 164.4 kg N ha^−1^ (Table 10) and for FI with 163.4 kg N ha^−1^ (Table 11). The bi-dimensional indicator ERG z-index showed for the overall dataset (Fig. s15) with 72.0 kg N ha^−1^ as the optimum N fertilization level (N_OPT_). W_W_ reached 89.4 kg N ha^−1^ at the N_OPT_ level and was higher than W_S_ with 40.0 kg N ha^−1^ (Table 11).

**Table 10:** Coefficient R^2^ for NEO, EUE and EP with coefficient of the second order polynomial for N fertilization. Estimated N fertilization level (N_OPT_) for NEO.

	R^2^	N_OPT_	Intercept	Linear	Quadratic
NEO (GJ ha^a^)			NEO	NEO	NEO
W_W_-2019/20	0.646∗∗	166.4	140.230	0.802^ab^	−0.002425
W_S_-2019/20	0.297	148.1	110.054	0.315^a^	−0.001144
W_W_-2020/21	0.704∗∗	158.3	147.025	1.122^b^	−0.003812
W_S_-2020/21	0.405∗	168.4	135.999	0.414^a^	−0.001603
Mean		160.2 (164.4)^a^			
EUE_GRAIN_ (GJ GJ^−1^)			EUE_GRAIN_	EUE_GRAIN_	EUE_GRAIN_
W_W_-2019/20	0.813∗∗∗		15.271^b^	−0.003	0.000004
W_S_-2019/20	0.949∗∗∗		12.446^ab^	−0.055	0.000101
W_W_-2020/21	0.682∗∗		12.583^ab^	−0.018	−0.000053
W_S_-2020/21	0.935∗∗∗		11.537^a^	−0.065	0.000161
EUE_AGB_ (GJ GJ^−1^)			EUE_AGB_	EUE_AGB_	EUE_AGB_
W_W_-2019/20	0.824∗∗∗		30.031	−0.076	0.000039
W_S_-2019/20	0.932∗∗∗		23.890	−0.106	0.000204
W_W_-2020/21	0.755∗∗∗		28.303	−0.023	−0.000174
W_S_-2020/21	0.918∗∗∗		26.178	−0.096	0.000132
EP_GRAIN_ (kg MJ^−1^)			EP_GRAIN_	EP_GRAIN_	EP_GRAIN_
W_W_-2019/20	0.813∗∗∗		0.936^b^	−0.002	2.4762E^−7^
W_S_-2019/20	0.949∗∗∗		0.763^ab^	−0.003	0.000006
W_W_-2020/21	0.682∗∗		0.771^ab^	−0.001	−0.000003
W_S_-2020/21	0.935∗∗∗		0.707^a^	−0.004	0.000010
EP_STRAW_ (kg MJ^−1^)			EP_STRAW_	EP_STRAW_	EP_STRAW_
W_W_-2019/20	0.821∗∗∗		0.925	−0.003	2.4762E^−7^
W_S_-2019/20	0.894∗∗∗		0.717	−0.003	0.000006
W_W_-2020/21	0.758∗∗∗		0.985	−0.003	−0.000008
W_S_-2020/21	0.823∗∗∗		0.917	−0.002	−0.000002
EP_AGB_ (kg MJ^−1^)			EP_AGB_	EP_AGB_	EP_AGB_
W_W_-2019/20	0.824∗∗∗		1.861	−0.005	0.000002
W_S_-2019/20	0.931∗∗∗		1.480	−0.007	0.000013
W_W_-2020/21	0.755∗∗∗		1.756	−0.001	−0.000011
W_S_-2020/21	0.917∗∗∗		1.624	−0.006	0.000008

a The mean value just for significant interactions is in bracket. Significance level for R^2^: p < 0.05 (∗), p < 0.01 (∗∗), p < 0.001 (∗∗∗).

**Table 11:** R^2^ for EI, FI and ERG z-index with coefficient of the second order polynomial for N fertilization.

	R^2^	N_OPT_	Intercept	Linear	Quadratic
EI_GRAIN_ (MJ kg^−1^)			EI_GRAIN_	EI_GRAIN_	EI_GRAIN_
W_W_-2019/20	0.825∗∗∗		1.077	0.002	0.000009
W_S_-2019/20	0.919∗∗∗		1.329	0.005	0.000016
W_W_-2020/21	0.696∗∗		1.324	0.001	0.000025
W_S_-2020/21	0.833∗∗∗		1.426	0.010	4.8232E^−8^
EI_AGB_ (MJ kg^−1^)			EI_AGB_	EI_AGB_	EI_AGB_
W_W_-2019/20	0.834∗∗∗		0.541	0.001^ab^	0.000005
W_S_-2019/20	0.885∗∗∗		0.683	0.003^b^	0.000006
W_W_-2020/21	0.805∗∗∗		0.581	0.000^a^	0.000010
W_S_-2020/21	0.942∗∗∗		0.618	0.002^b^	0.000006
Allocated EI_GRAIN_ (MJ kg^−1^)			Allocated EI_GRAIN_	Allocated EI_GRAIN_	Allocated EI_GRAIN_
W_W_-2019/20	0.832∗∗∗		0.272^a^	0.000775	0.000002
W_S_-2019/20	0.836∗∗∗		0.352^b^	0.001663	0.000002
W_W_-2020/21	0.828∗∗∗		0.255^a^	−0.000206	0.000004
W_S_-2020/21	0.894∗∗∗		0.271^a^	0.000199	0.000005
Allocated EI_STRAW_ (MJ kg^−1^)			Allocated EI_STRAW_	Allocated EI_STRAW_	Allocated EI_STRAW_
W_W_-2019/20	0.825∗∗∗		0.269	0.001	0.000002
W_S_-2019/20	0.919∗∗∗		0.332	0.001	0.000004
W_W_-2020/21	0.731∗∗∗		0.326	0.000097	0.000006
W_S_-2020/21	0.888∗∗∗		0.347	0.002	8.065E^−7^
FI (L 100 kg^−1^)			FI	FI	FI
W_W_-2019/20	0.760∗∗∗	171.2	1.291	−0.006	0.000017
W_S_-2019/20	0.437∗	163.6	1.584	−0.004	0.000014
W_W_-2020/21	0.510∗	154.3	1.419	−0.007	0.000024
W_S_-2020/21	0.029	62.3	1.531	0.000	−0.000001
Mean		137.8 (163.0)^a^			
ERG z-index					
W_W_-2019/20	0.108	83.3	−0.108	0.011497	−0.000069
W_S_-2019/20	0.201	4.0	0.526	0.000296	−0.000037
W_W_-2020/21	0.267	93.1	−0.494	0.025702	−0.000138
W_S_-2020/21	0.256	57.2	0.289	0.009147	−0.000080
W_W_	0.179 (p = 0.07)	89.4	0.209	0.005897	−0.000053
W_S_	0.222∗	40.0	0.017	0.017424	−0.000109
2019/20	0.130	55.6	0.209	0.005897	−0.000053
2020/21	0.228	79.9	0.017	0.017424	−0.000109

a The mean value just for significant interactions is in bracket. Significance level for R^2^: p < 0.05 (∗), p < 0.01 (∗∗), p < 0.001 (∗∗∗).

The energy efficiency indicators EUE and EP decreased with N fertilization level, and the energy efficiency EI indicators increased with N fertilization level in the range between 0 and 200 kg N ha^−1^.

The overall estimated values for the dependent variables in the control were for NEO: 133.3 GJ ha^−1^, EUE_GRAIN_: 13.0 GJ GJ^−1^, EUE_AGB_: 27.1 GJ GJ^−1^, EI_GRAIN_: 1.29 MJ kg^−1^, EI_AGB_: 0.61 MJ kg^−1^, allocated EI_GRAIN_: 0.29 MJ kg^−1^, allocated EI_STRAW_: 0.32 MJ kg^−1^, EP_GRAIN_: 0.79 kg MJ^−1^, EP_STRAW_: 0.89 kg MJ^−1^, EP_AGB_: 1.68 kg MJ^−1^, FI: 1.46 L 100 kg^−1^.

## 4 Discussion

### 4.1 Total fuel consumption and total energy input

Intensification in plant production is often linked with higher area-based fuel consumption, especially if the intensification takes place with more tillage operations, e.g. with conventional tillage using a mouldboard plow [38]. In our study, the total area-based fuel consumption was lower than 60 L ha^−1^, which is representative of wheat production in a reduced tillage system without mouldboard plow [21,36] in the Pannonian region. Shifting tillage systems from conventional using a mouldboard plow to plowless tillage systems reduces not only direct energy input but also the working time [36]. These benefits are the main economic reasons why plowless tillage systems are increasingly practiced in the Pannonian region. In dry climate regions, crop yields of conservation tillage systems (e.g. no-till) can be equivalent or greater compared to conventional tillage systems. This suggests that plowless conservation tillage may become an important adaptation strategy adaptation to climate change in drier regions of the world [39]. However, the energy savings of conservation tillage are often offset by higher energy requirements for herbicides and nitrogen fertilizers [7].

Only 2.5 % of the total fuel consumption is accounted for the one-pass spreading of mineral N fertilizer. In contrast to spreading mineral fertilizer, the fuel consumption of fertilization with farmyard manure, compost, or slurry is much higher [38], which is explained by the lower nutrient density in organic manure than in solid mineral fertilizer. For the application of 50 kg N, about 108 kg of mineral N fertilizer (Urea, 46%N) or about 13 000 kg of cattle farmyard manure (3.2 kg N m^−3^) are required. This is often the reason for the higher fuel consumption for fertilization in organic than conventional farming systems [40].

A lower consumption of mineral fertilizers results in a lower energy consumption [41]. In contrast to fuel consumption, the indirect energy input through mineral N fertilizer, which is consumed during its production, affected the total energy input significantly. This reveals the importance of mineral N fertilizer in energy efficiency analyses, which is also reported in other studies [19,27]. The sowing times of facultative wheat do not affect the area-based fuel consumption as well as the indirect energy input if the seed amount is the same.

The area-based total energy input ranged between 5.0 GJ ha^−1^ in the zero N fertilization treatment to 13.5 GJ ha^−1^, which is mainly caused by the amount of mineral N fertilization. The area-based total energy input is an indicator of the production intensity of cropping systems [18]. In some low-input arable farming systems, e.g. in large areas of Africa, the energy input is lower than 1 GJ ha^−1^, whereas, in some modern high-input farming systems in the USA and Western Europe, it can exceed 30 GJ ha^−1^ [40,42]. In Europe, there is a wide range of the area-based energy input, which depends on pedo-climatic region (Alpine, Boreal, Nemoral, Continental, Atlantic Central, Pannonian, Lusitanian, Anatolian, Mediterranean) and farming system (low-input systems <10 GJ ha^−1^: extensive grassland, organic arable farming; high-input systems >10 GJ ha^−1^: intensive conventional farming, horticulture, greenhouse cultivation) [40]. In our study, the threshold (10 GJ ha^−1^) between a low-input system and a high-input system is reached at the mineral N fertilization of about 117 kg N ha^−1^ (a result of the linear interpolation between 100 kg N ha^−1^ and 150 kg N ha^−1^).

### 4.2 Crop yield and energy output

Crop yield and energy output are important parameters for the assessment of cropping systems. This is of high importance, as the availability of arable land is limited, especially in many densely populated areas in the world [18].

Area-based crop yield end energy output (EO) are indicators that reveal the photosynthetic performance of a crop in a certain pedo-climate region under agronomic management. In the Pannonian region, with its semi-arid climate conditions, water is the limiting factor for plant growth and is mainly responsible for the yield variability of agricultural crops [43]. Long-term experiments in the Pannonian climate region showed that environmental factors influence grain yield and energy efficiency more strongly than management practices (e.g. tillage) [21]. The timing of field operations (e.g. tillage, fertilization) is a crucial management constraint responsible for crop yield stability in a certain growing season [8].

Drought is a relevant cause of low yields worldwide. Therefore, there is an urgent need for more water efficient cropping systems with low unproductive losses via runoff and evaporation [43]. Conservation tillage in the Pannonian region improved water supply to the crop, associated with higher soil water storage capacity and a decrease of unproductive water losses [44]. Our study shows that also the sowing time of wheat is a management factor which affects the crop yield and EO positively. Overall, the crop yield and EO were for W_W_ between 25.0 % and 47.5 % higher than for W_S_, which is explained by the extension of the vegetation period and better fitting of the water demand of the plants to soil water supply (better water usage of the winter precipitation) [43].

The effect of mineral N fertilization rates on the energy efficiency indicators EO_GRAIN_, EO_STRAW_, EO_AGB_ and NEO was small, indicating that the additional fertilized N was only partly transformed into biomass. The estimated optimal N fertilization for maximum EO_AGB_ with 177.7 kg N ha^−1^ is very high, which is, from the ecological point of view (soil N accumulation and N emission of N_2_O and NO_3_-N), not recommended for this site. High amounts of N can be lost at high N fertilization rates. A previous N efficiency analysis at the same site showed that a one dose application of 160 kg N ha^−1^ with urea (46 %, ALZON 46®) resulted just in an apparent N recovery efficiency of 47 % [25]. The N efficiency and also the crop yield can be increased by splitting the N fertilizer application into two or three doses during the vegetation period, but this also causes a higher area-based fuel consumption and a higher energy input [5].

The optimizationof the uptake of the fertilized mineral N by the crop is key for increasing crop yield and EO, as well as the energy efficiency indicators in semi-arid regions. Irrigation could be the technical solution for increasing N efficiency with increased crop yield and EO. Due to economic issues, irrigation of winter wheat was not usual in previous times. Spring wheat, especially spring durum wheat (*Triticum durum* L.), will be irrigated during dry vegetation periods. A previous energy efficiency analysis at the same site in the Pannonian region showed that irrigation of winter wheat increased crop yield and EO significantly [45].

### 4.3 Energy efficiency

The energy efficiency indicators NEO, EUE, EI, EP and FI allow a deeper insight into the energetic situation of a cropping system. These indicators are mainly determined by the higher crop yield as well as energy output in comparison to the lower energy input values, as already indicated in former studies [27,40]. Eventually, crop yield is an important determinant of energy efficiency in plant production.

NEO describes the energy gain [46] and is the most relevant parameter in determining the efficiency of cropping systems in a world of increasing food and energy demand [18,27]. From this point of view, W_W_ performed better than W_S_, whereas N fertilization did not affect NEO between 50 and 200 kg N ha^−1^. NEO, as well as crop yield and EO, respond to mineral N fertilization according to the Mitscherlich effect law in plant production, which postulates that yield increments decrease with increasing N fertilization [47]. The estimated N fertilization level (N_OPT_) for the highest NEO with 160.2 kg N ha^−1^ (Table 10) is high for crop yield and EO.

EUE shows the ratio of energy output to energy input and is one of the best energy indices for indicating the efficient use of energy [26]. It is also used for determining the optimum input levels from an ecological point of view [18,26]. In our study, EUE_Grain_ and EUE_AGB_ were significantly higher in W_W_ than W_S_ and decreased significantly with increasing mineral N fertilization. From the ecological point of view, zero N fertilization resulted in the highest EUE_Grain_ and EUE_AGB_.

EI or specific energy [26] indicates the amount of energy used to produce 1 kg of crop (grain or straw). EP is the inverse of EI. A higher EP is realized if more crop yield (grain or straw) is produced per unit of energy input. In all EI and EP indicators in our study, W_W_ performed better than W_S,_ and with increasing mineral N fertilization the EP indicators get worse. EI and EP are indicators for finding the optimum input level for optimum crop management intensities. From this point of view, zero N fertilization resulted in the best values of EI and EP. But these uni-dimensional indicators as well as the EUE do not consider the necessary arable area for crop production. Multi-dimensional indicators, as recommended by Rossner et al. [35], capture important features of production efficiency and are more objective as advisory tools for sustainable crop production. The bi-dimensional indicator EGR z-index, a z-score approach that combines NEO and EUE, showed an overall optimum N level with 72.0 kg N ha^−1^. This estimated N level is about 40 % lower than the N level recommended by the national guideline for fertilization for cropping and grassland [48]. Rainfed wheat with this fertilization management belongs to low-input systems with lower than 10 GJ ha^−1^ energy input [40].

In a long-term field experiment on a sandy loam Stagnic Albeluvisol in Estonia, the optimum N fertilization level for spring wheat was estimated with the EGR z-index at 95 kg N ha^−1^, whereas the EUE indicated the optimum level at 66 kg N ha^−1^ and the NEO at 119 kg N ha^−1^ [35].

Compared to organically fertilized cropping systems, the energy efficiency indicators are better than in NPK fertilized treatments [42,49,50]. However, an energetic consideration of the mineral nutrients in the organic manure of organically fertilized cropping systems resulted in worse energy efficiency parameters but nevertheless were better than in NPK-fertilized cropping systems [38].

Overall, the results of the present study indicated that W_W_ is more energy efficient than W_S_. From this point of view W_W_ is more suitable than W_S_ in the Pannonian region.

N fertilization with the one-dose application of urea has a limited effect on energy efficiency improvement. Consequently, there exists an enormous potential exists for increasing the energy efficiency during mineral N fertilization in the Pannonian region. The ongoing climate change will affect the energy efficiency indicators negatively if no adaptation measurements are done [51].

## 5 Conclusions

In the semi-arid Pannonian region, where soil water content and precipitation mainly determine plant growth, the sowing time of wheat and also the mineral N fertilization level play an important role in the energy efficiency of arable farming.

The effect of mineral N fertilization under Pannonian climate conditions was low, which is mainly explained by the application regime (one-dose application). The energy efficiency indicator NEO, which shows the area-based energy gain, was estimated highest with 160.2 kg N ha^−1^, which is from the point of soil N accumulation and potential N emission (N_2_O into the atmosphere and NO_3_^−^-N leaching into the aquifer) not sustainable for this pedo-climatic region. From the ecological point of view, zero N fertilization performed best, as the indicators EUE, EI, and EP showed. A multi-dimensional z-score approach allows in the N optimization task a better balance between the crop area orientated indicator (NEO) and crop yield orientated indicator (EUE). According to this approach, an optimal N fertilization level was estimated at 89.4 kg N ha^−1^ for W_W_ and 40.0 kg N ha^−1^ for W_S_.

The matching of the N demand by crops during the vegetation period with the mineral N supply is key for increasing the energy efficiency indicators, where a high NEO could be realized with high EUE and EP and low EI.

## Funding

This work was supported by the project “DiLaAg – Digitalization and Innovation Laboratory in Agricultural Sciences” of the private foundation “Forum Morgen, Austria” and the Federal State of Lower Austria.

## Data availability statement

Data included in article/supplementary material in the article.

## CRediT authorship contribution statement

**Gerhard Moitzi:** Writing – review & editing, Writing – original draft, Visualization, Validation, Software, Methodology, Investigation, Formal analysis, Data curation, Conceptualization. **Lukas J. Koppensteiner:** Writing – review & editing, Methodology, Investigation, Data curation, Conceptualization. **Agnieszka Klimek-Kopyra:** Writing – review & editing, Supervision. **Jaroslav Bernas:** Validation, Supervision. **Hans-Peter Kaul:** Supervision, Resources, Funding acquisition. **Helmut Wagentristl:** Resources, Methodology. **Pia Euteneuer:** Resources, Project administration, Investigation. **Reinhard W. Neugschwandtner:** Writing – review & editing, Supervision, Resources, Project administration, Funding acquisition.

## Declaration of competing interest

The authors declare that they have no known competing financial interests or personal relationships that could have appeared to influence the work reported in this paper.
