# Supplementary material for: Effects of sowing date and nitrogen applications on the energy efficiency of facultative wheat (Triticum aestivum L.) in a Pannonian environment
Source: Heliyon. 2024 Sep 20;10(19):e37923. doi: 10.1016/j.heliyon.2024.e37923 (PMC11462256; doi:10.1016/j.heliyon.2024.e37923)
Supplement: Multimedia component 1 [file mmc1.docx]

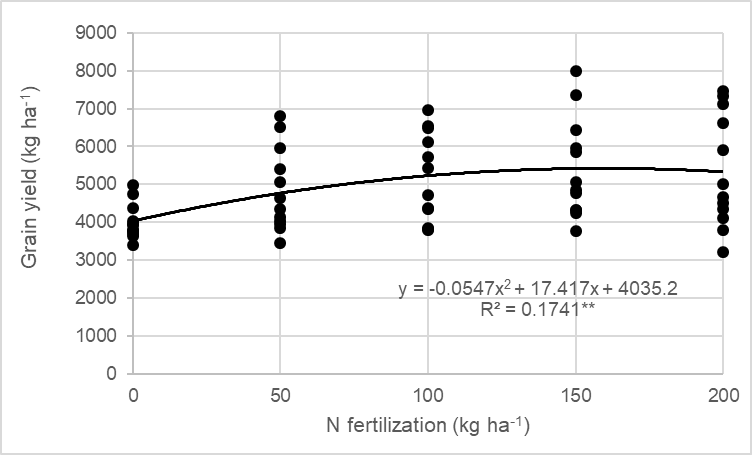


**Fig. s1.** Overall grain yield plotted against N fertilization rates.

**Fig. s2.** Overall straw yield plotted against N fertilization rates.


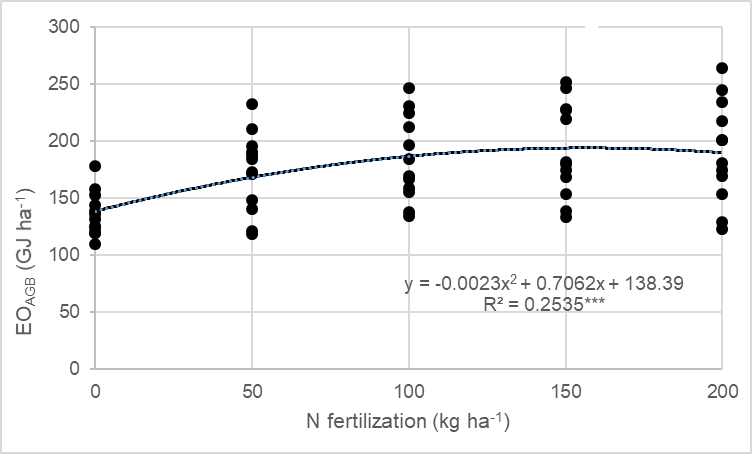


**Fig. s3:** Overall EO_AGB_ plotted against N fertilization rates.


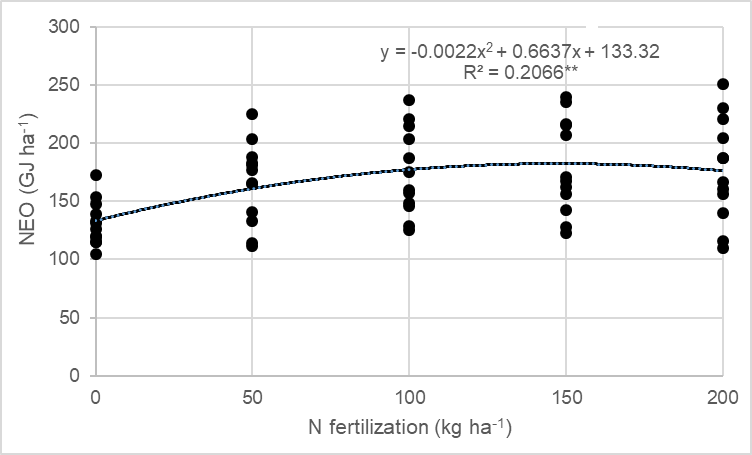


**Fig. s4**. Overall NEO plotted against N fertilization rates.


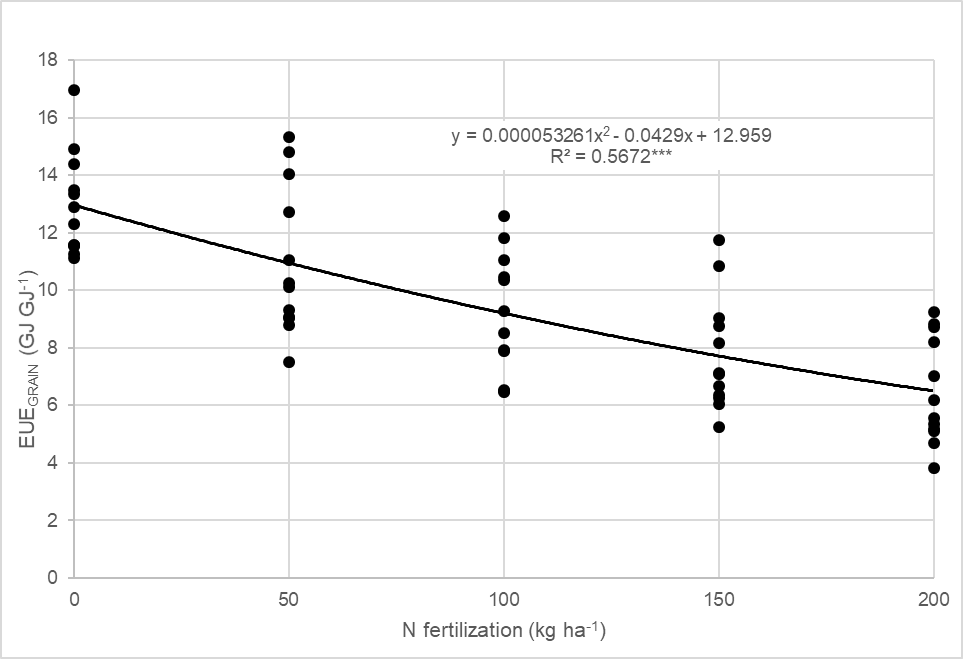


**Fig. s5**. Overall EUE_GRAIN_ plotted against N fertilization rates.


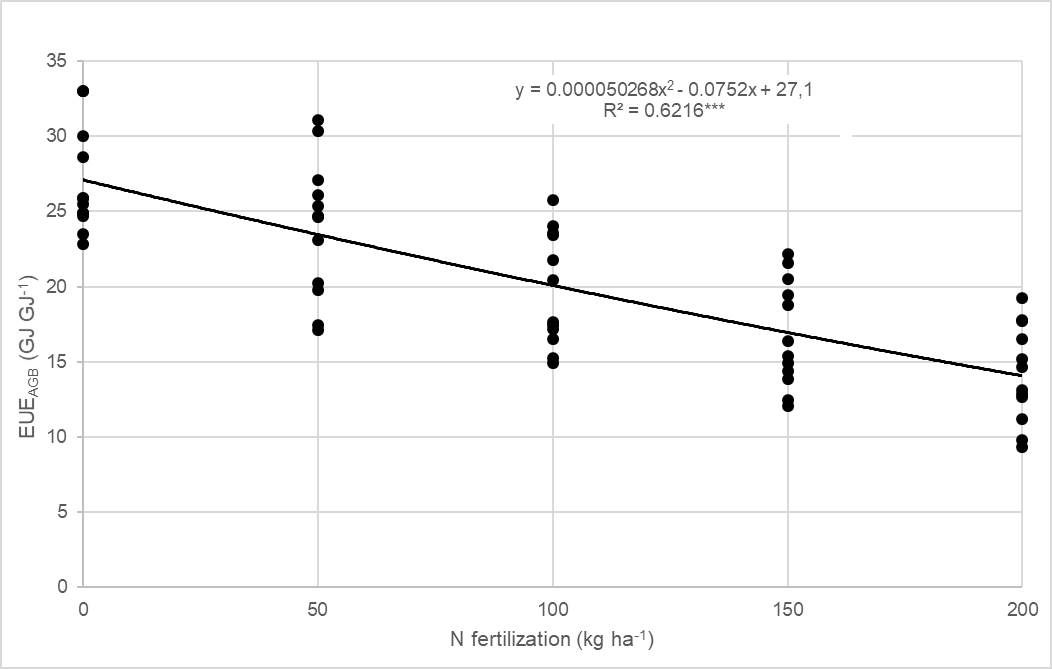


**Fig. s6.** Overall EUE_AGB_ plotted against N fertilization rates.


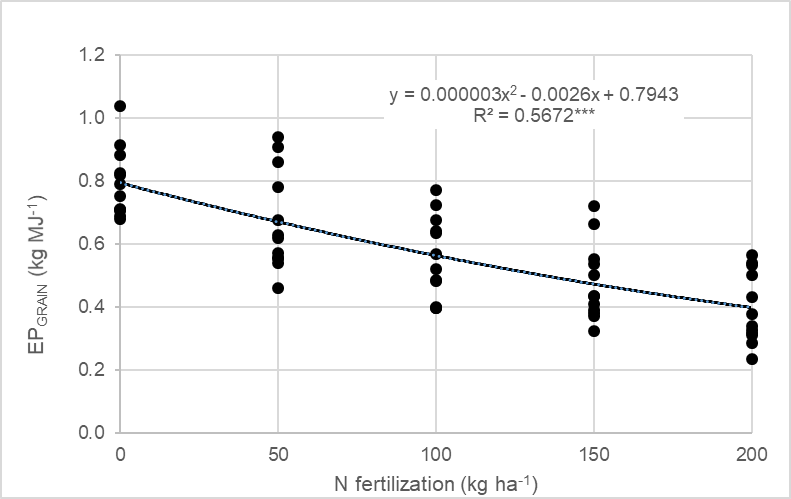


**Fig. s7.** Overall EP_GRAIN_ plotted against N fertilization rates.

**Fig. s8.** Overall EP_STRAW_ plotted against N fertilization rates.

**Fig. s9.** Overall EP_AGB_ plotted against N fertilization rates.


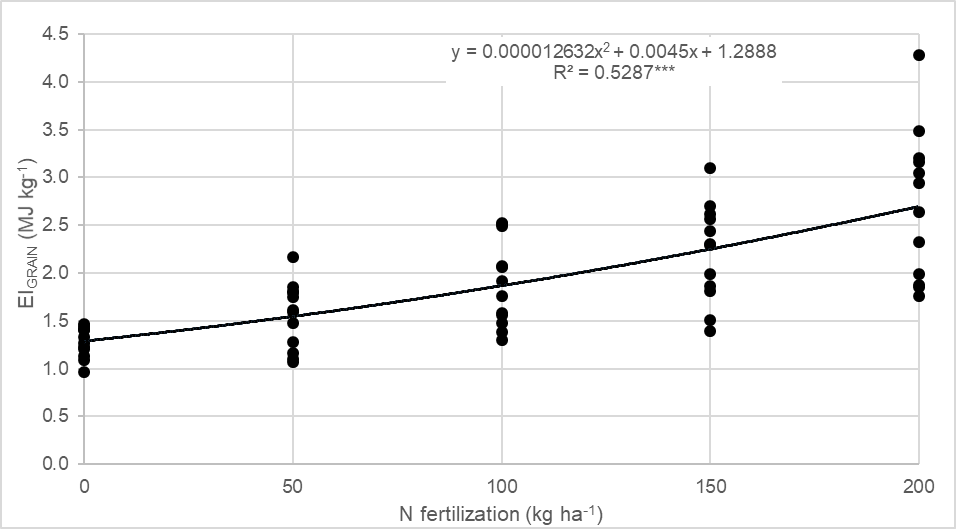


**Fig. s10.** Overall EI_GRAIN_ plotted against N fertilization rates.


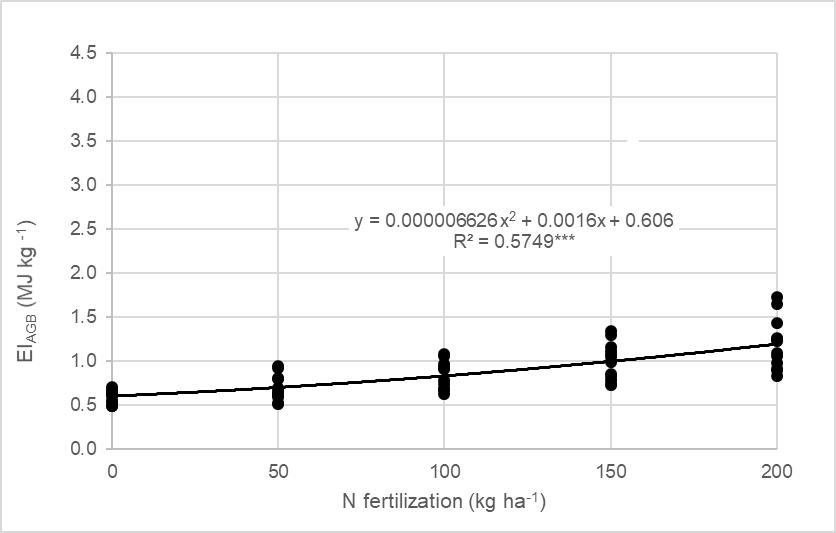


**Fig. s11:** Overall EI_AGB_ plotted against N fertilization rates.


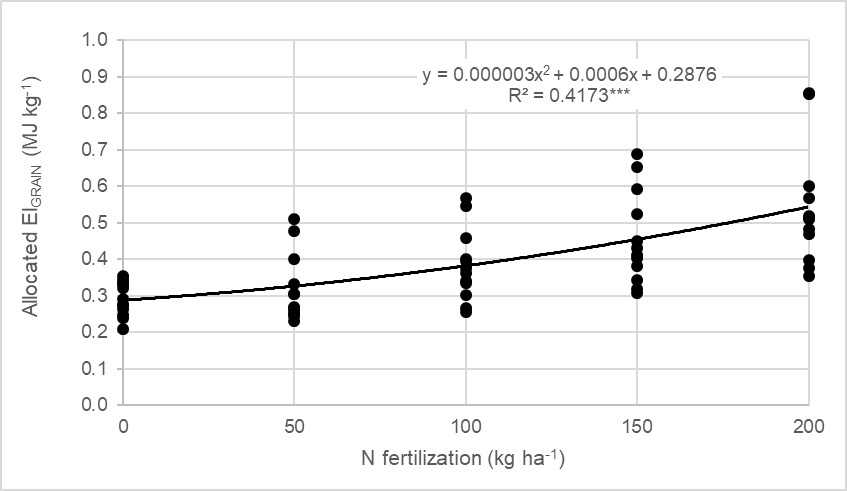


**Fig. s12.** Overall allocated EI_GRAIN_ plotted against N fertilization rates.


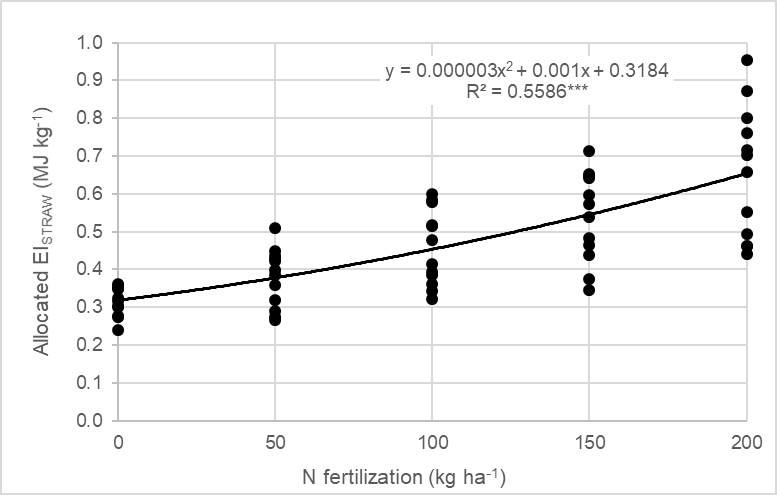


**Fig. s13.** Overall allocated EI_STRAW_ plotted against N fertilization rates.


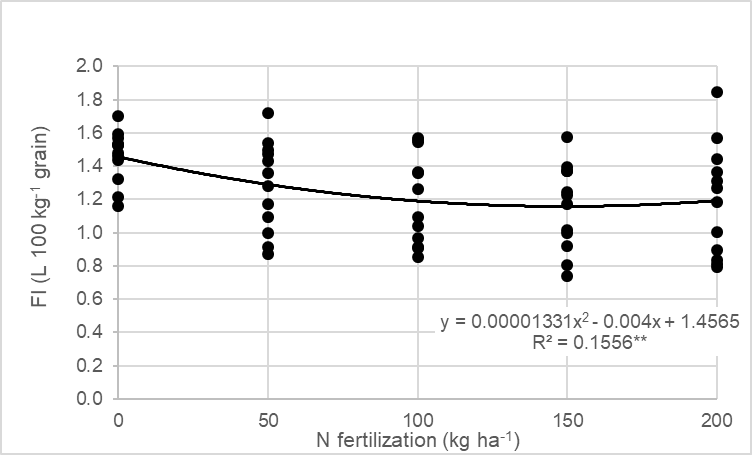


**Fig. s14.** Overall FI plotted against N fertilization rates.


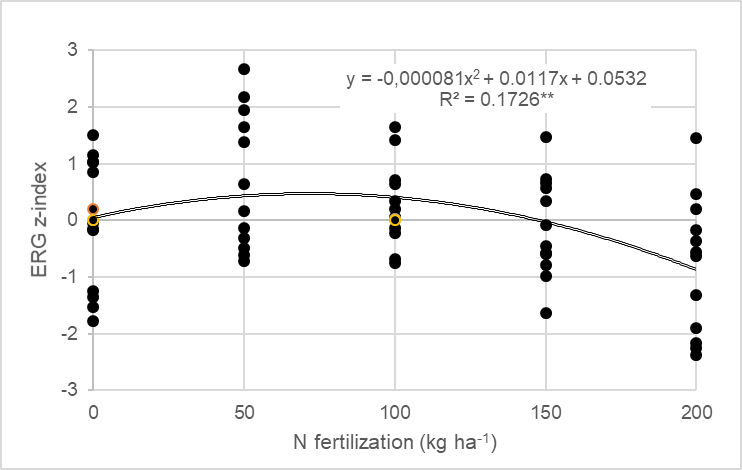


**Fig. s15.** Overall ERG z-index plotted against N fertilization.
